# Serum microRNA-205 as a novel biomarker for cervical cancer patients

**DOI:** 10.1186/s12935-014-0081-0

**Published:** 2014-08-22

**Authors:** Quanhui Ma, Guiping Wan, Shuxia Wang, Wanwei Yang, Jiaming Zhang, Xiaoming Yao

**Affiliations:** 1 Department of clinical laboratory, Jiangsu Province hospital on Integration of Chinese and Western Medicine, Nanjing University of Traditional Chinese Medicine, Jiangsu Branch of China Academy of Chinese Medical Science, Nanjing 210028, China; 2 Department of obstetrics and gynecology, Jiangsu Province Hospital on Integration of Chinese and Western Medicine, Nanjing, China

**Keywords:** Cervical cancer, Serum miR-205, Diagnosis, Prognosis, Biomarker

## Abstract

**Objective:**

Serum microRNAs (miRNAs) are a novel class of diagnostic and prognostic biomarkers for numerous cancers. However, the level and clinical relevance of circulating miR-205 transcripts in human serum of cervical cancer patients are unclear. The purpose of this study was to determine serum miR-205 levels in cervical cancer patients and explore its association with clinicopathological factors and prognosis.

**Methods:**

Serum miR-205 expression was investigated in 60 cervical cancer patients and 60 healthy normal controls by using real-time PCR. Correlations between miR-205 expression and the clinicopathological features and prognosis of cervical cancer patients were then evaluated. Receiver operating characteristic curves were used to evaluate the sensitivity and specificity of serum miR-205.

**Results:**

Serum miR-205 was significantly upregulated in cervical cancer patients compared with healthy donors (p < 0.01), and a high level of miR-205 expression was correlated with poor tumor differentiation (p = 0.009), lymph node metastasis (p = 0.015) and increased tumor stage (p = 0.001). The serum miR-205 level was capable of separating advanced stage from early stage metastatic cervical cancer from non-metastatic samples and poorly differentiated tumors from differentiated tumors with an area under the curve values of 0.74, 0.694 and 0.717, respectively. The expression of miR-205 was also higher in the cervical cancer tissues compared with the para-carcinoma tissues. In addition, Kaplan-Meier survival analysis showed that cervical cancer patients with high miR-205 expression tended to have shorter overall survival. In multivariate Cox regression analysis, miR-205 was identified as an independent prognostic marker.

**Conclusions:**

Serum miR-205, which is upregulated in cervical cancer, represents a predictive biomarker for the prognosis of cervical cancer patients.

## Introduction

Cervical cancer, one the most commonly malignant tumors, has become a major health problem for women worldwide, particularly in developing countries [1],[2]. Cervical cancer develops step-by-step and involves a sequential progression from normal cervical epithelium to preneoplastic cervical intraepithelial neoplasia and then to invasive cervical cancer [3]. Increasing evidences showed that early detection by testing for high-risk human papillomavirus (HPV) and cervical papilloma smears have reduced cervical cancer mortality. However, these methods do not detedted the development of cervical cancer directly [4]. Therefore, new and less invasive biomarkers are needed to improve the detection and prognostic outcome of cervical cancer.

MicroRNAs (miRNAs), small non-coding RNA molecules, play a central role in post-transcriptional gene regulation by binding to a target site in the 3’-UTR of target mRNAs [5]. MiRNAs are involved in pathological and physiological activities [6]. In recent years, the relationship between miRNA and cancer has become a research focus. Tumor initiation and progression is related to altered expression of miRNAs [7]. MiRNAs are not only expressed in cancer tissues but also in sera [8],[9]. Further investigation confirmed that circulating miRNAs can originate from cancer tissues and are highly stable in serum and plasma [10]. As a result, circulating miRNAs have been exploited as a diagnostic tool for early cancer detection, risk assessment and prognosis [11].

MiR-205 acts as an oncogene by modulating the expression of multiple cancer-related target genes [12]. In addition, miR-205 is significantly overexpressed in human cervical cancer tissues and promotes proliferation and migration of cervical cancer cells by targeting CYR61 and CTGF [13]. Circulating miR-205 has been reported as a biomarker for the detection and diagnosis of lung cancer, particularly at its very early stage [14]. However, the clinical significance of circulating miR-205 levels in cervical cancer remains unclear. Thus, the aim of this study was to evaluate whether serum miR-205 was capable of acting as a diagnostic and prognostic biomarker for cervical cancer patients. Here, we report that serum miR-205 is upregulated in cervical cancer and represents a predictive biomarker for the prognosis of cervical cancer patients.

## Results

### Higher levels of miR-205 in the serum of cervical cancer patients

**Figure 1:**
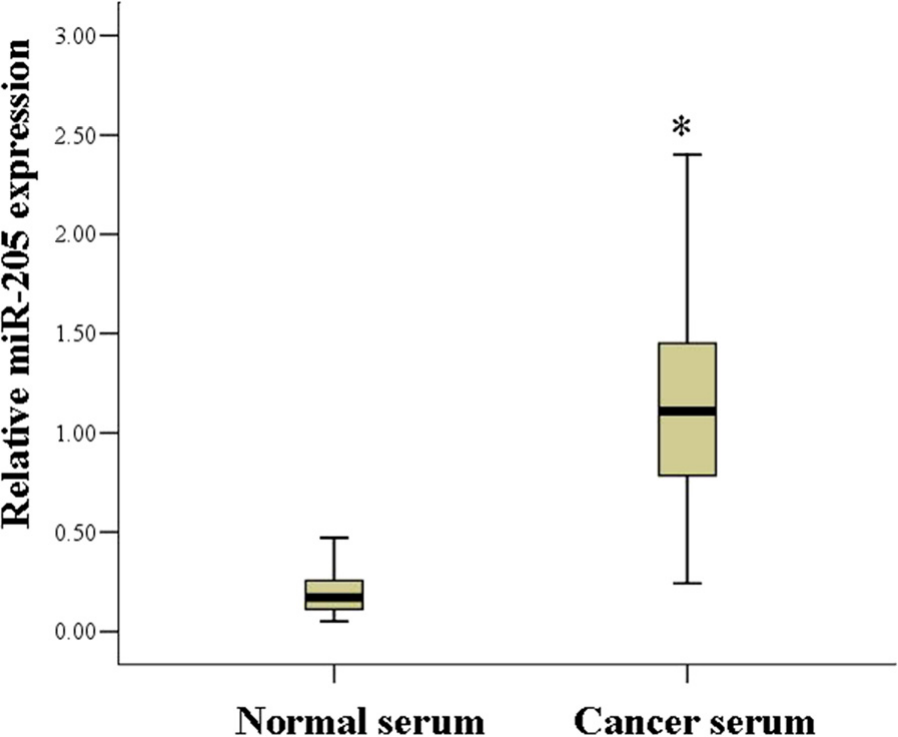
**Expression analysis of miR-205 in the serum of cervical cancer patients.** The expression of miR-205 in cervical cancer patient serum was higher that in normal controls (P < 0.01).

MiR-205 expression was significantly higher in human cervical cancer than in normal tissue, and it also promotes cervical cancer cell proliferation and migration [13]. Therefore, the level of miR-205 was determined in the serum of the cervical cancer patients. The results showed that among the 60 cervical cancer samples analyzed, serum miR-205 was upregulated 5.74-fold in cervical cancer patients compared with the level in healthy donors. Summarized data from all individuals indicated that the relative expression of miR-205 in cervical cancer patients’ serum (1.18 ± 0.57) was significantly higher than that in healthy donors’ serum (0.2 ± 0.12) (P < 0.01, Figure 1).

### Higher expression of miR-205 in cervical cancer tissues

**Figure 2:**
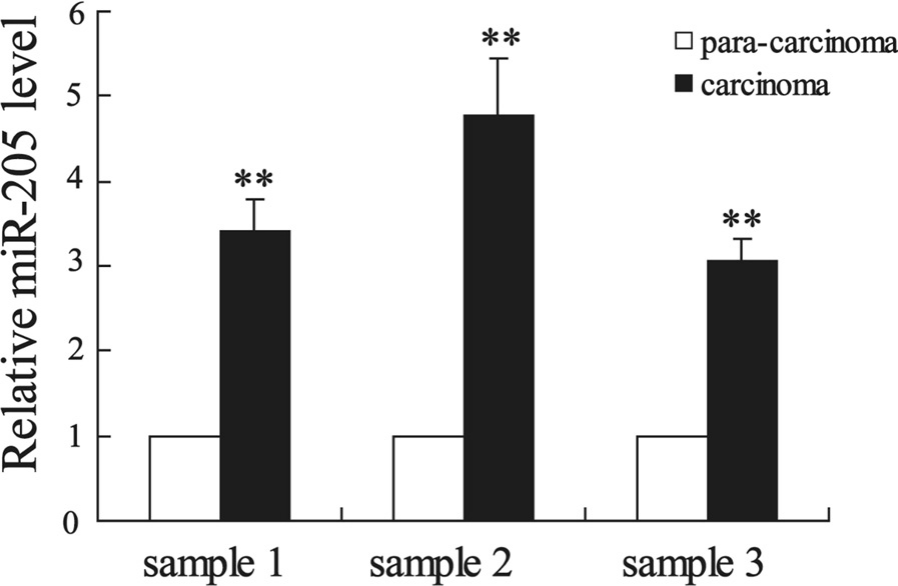
**The expression of miR-205 in cervical cancer tissues.** qPCR results showed that miR-205 expression was considerably higher in the cervical cancer tissues compared with para-carcinoma tissues (**P < 0.01).

To further demonstrate the role of miR-205 in cervical cancer, its expression was measured in cervical cancer tissues. As shown in Figure 2, the miR-205 expression was significantly increased (>3-fold higher) in cervical cancer tissues (n = 3) compared with paracancerous tissues (n = 3) (p < 0.01), which is consistent with an oncogenic role of miR-205.

### Correlation of clinicopathological features of cervical cancer with circulating miR205

To better understand the potential roles of serum miR-205 in cervical cancer development and progression, the relationships between miR-205 and various clinical features of cervical cancer were determined. In the present study, the average fold change of miR-205 (miR-205 in serum of cervical cancer patient compared with healthy donors) was 5.74-fold. The average fold change was used as the threshold, and patients were separated into high expression (above 5.74-fold) and low expression (below 5.74-fold) groups. As shown in Table 1, miR-205 expression was significantly higher in the serum of patients with advanced FIGO stage cervical cancer than those with early FIGO stage (P = 0.001, Table 1). The expression of miR-205 in lymph node metastasis-positive patients was significantly increased compared to that in lymph node metastasis-negative patients (P = 0.015, Table 1). There was a tendency for less well differentiated tumors to express higher levels of miR-205 (P = 0.009, Table 1). However, there was no correlation between miR-205 expression and other clinical features, such as age and HPV infection.

**Table 1:** Correlation of clinicopathological features of cervical cancer with serum miR-205 expression levels

**Characteristics**	**All cases**	**Serum miR-205**		
		**High expression**	**Low expression**	**P value**
Age				0.796
<50	28	15	13	
≥50	32	15	17	
Tumor size (cm)				
<4	32	14	18	0.438
≥4	28	16	12	
HPV				0.671
Positive	54	28	26	
Negative	6	2	4	
Differentiation				0.009
Well-moderately differentiated	27	8	19	
Poor differentiation	33	22	11	
FIGO stage				0.001
Ib ~ IIa	26	6	20	
IIb ~ IIIa	34	24	10	
Lymph node metastasis				0.015
No	22	6	16	
Yes	38	24	14	

### Serum miR-205 correlates with prognosis of cervical cancer patients

**Figure 3:**
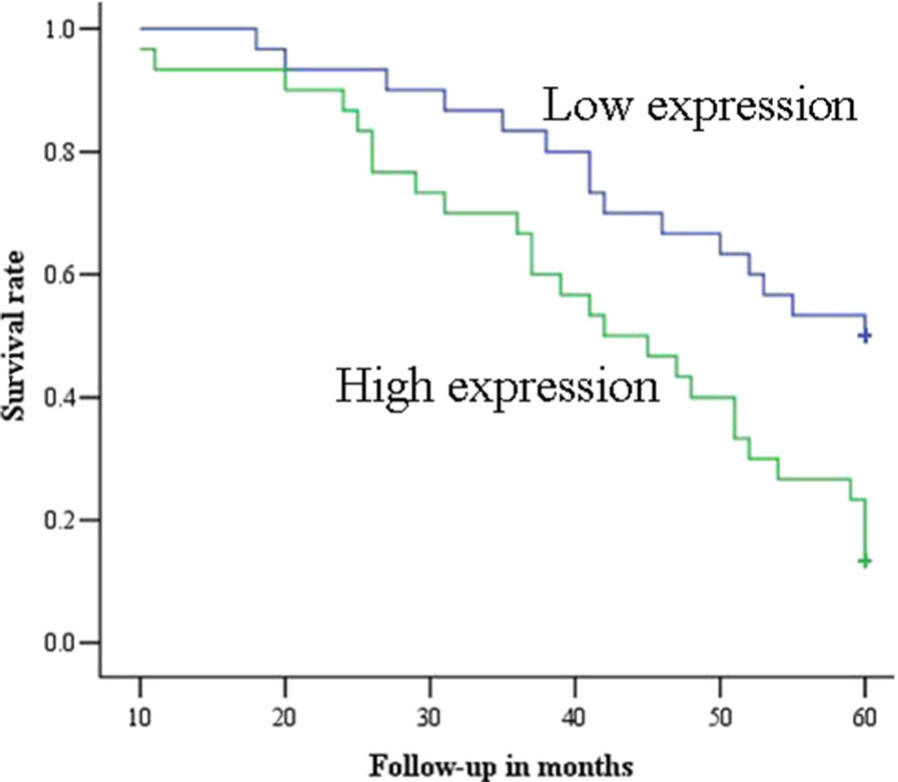
**Kaplan-Meier survival curves of cervical cancer patients.** The 5-year overall survival rate of cervical cancer patients with high serum miR-205 expression (16.67%) was significantly lower than that of cervical cancer patients with low serum miR-205 expression (53.33%, P = 0.003).

To further evaluate whether serum miR-205 levels were associated with cervical cancer prognosis, we performed survival analysis. The survival period of the patients was defined as the duration from the time of surgery to death, or to the last follow-up day. As shown in Figure 3, cervical cancer patients with higher miR-205 expression had significantly poorer survival than those with lower expression of miR-205 (log-rank test: P = 0.003). The 5-year overall survival rate in cervical cancer patients with lower serum miR-205 expression was 53.33%, whereas that of patients with higher miR-205 expression was 16.67%. To determine the possibility of serum miR-205 as an independent risk factor for poor prognosis, both clinicopathological factors and the level of serum miR-205 expression were evaluated by multivariate Cox regression analysis. The results showed that lymph node metastasis and high level of serum miR-205 were independent factors to predict the overall survival of cervical cancer patients (Table 2).

**Table 2:** Multivariate analysis for prognostic factors

**Variate**	**Subset**	**Relative risk (95% CI)**	**P value**
Age (year)	<50/≥50	0.661 (0.294-1.484)	0.315
Tumor size (cm)	<4/≥4	1.341 (0.616-3.919)	0.460
Tumor differentiation	Well-moderately/poor	0.836 (0.057-12.347)	0.896
FIGO stage	Ib ~ IIa/IIb ~ IIIa	0.577 (0.038-8.702)	0.691
Lymph node metastasis	Yes/no	0.032 (0.003-0.332)	0.004*
miR-205	Low/high	3.011 (1.312-6.910)	0.009*

95% CI: 95% confidence interval. *P < 0.05.

### Capability of miR-205 to function as a biomarker for cervical cancer prognosis

**Figure 4:**
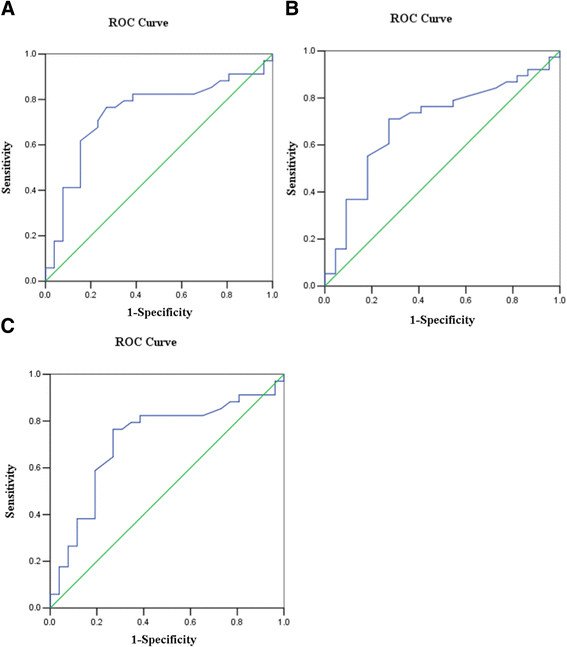
**Receiver operating characteristic (ROC) analysis was performed to determine the sensitivity and specificity of the miR-205 expression level using area under the ROC curve (AUC) analysis. (A)** Separating stages Ib ~ IIa from stage IIb ~ IIIa. **(B)** Separating metastatic cervical cancer from non-metastatic samples. **(C)** Separating well to moderately differentiated from poorly differentiated.

The specificity and sensitivity of miR-205 as a cervical cancer prognosis biomarker was calculated by receiver operating characteristic (ROC) curves. ROC analysis revealed that miR-205 had a sensitivity of 76.5%, a specificity of 73.1% and an area under the curve (AUC) of 0.74 (p = 0.002, Figure 4A) when comparing stages Ib ~ IIa and stage IIb ~ IIIa. MiR-205 had an AUC of 0.694 with a sensitivity of 71.1% and specificity of 72.7% (p = 0.013, Figure 4B) for separation of metastatic cervical cancer from non-metastatic samples. ROC curve analysis also revealed that serum miR-205 was a valuable biomarker to distinguish well to moderately differentiated from poorly differentiation tumors, with a sensitivity of 76.5%, a specificity of 73.1% and an AUC of 0.717 (p = 0.004, Figure 4C). The cut-off value was 0.995 with the highest specificity and sensitivity. Taken together, this analysis revealed that serum miR-205 is a novel and efficient biomarker for cervical cancer prognosis.

## Discussion

Increasing evidence shows that serum miRNAs are promising novel biomarkers for the diagnosis and prognosis of cancer [8]. In this study, we first confirmed by qPCR that miR-205 levels are significantly higher in the serum of cervical cancer patients, and that a high level of miR-205 expression correlated with poor tumor differentiation, lymph node metastasis and increased tumor stage. Notably, patients with high serum miR-205 levels had a significantly lower survival rate than those with low expression levels, and serum miR-205 was an independent risk factor for poor prognosis. These results suggested that serum miR-205 could be used as a potential predictor of prognosis in cervical cancer.

MiR-205 is frequently dysregulated in many cancers and acts as a tumor suppressor or an oncogene depending on cellular context [15]. In cervical cancer, miR-205 functions as an oncogene, promoting proliferation and migration of cancer cells [13]. Our findings are suggestive of an oncogenic role for miR-205, with higher circulating expression of miR-205 in cervical cancer patients with a lower survival rate. In this study, we identified that miR-205 expression was significantly increased in cervical cancer tissues compared with paracancerous tissues. In addition, we confirmed, for the first time, that cervical cancer patients had significantly elevated miR-205 levels in their serum samples. This may be explained by miR-205 being released from cervical cancer cells into the peripheral blood, which is consistent with previous reports [15]. An increase in serum miR-205 may create a cancer- and metastasis-promoting environment. Thus, high levels of miR-205 expression were correlated with poor tumor differentiation, metastasis and increased tumor stage.

Tumor tissue miR-205 or serum miR-205 are associated with the development and prognosis of tumors [15]. Lebanony et al. reported that miR-205 in tissue samples is a highly accurate marker for distinguishing squamous from nonsquamous non-small-cell lung carcinoma [16]. Moreover, the downregulation of miR-205 expression in colorectal cancer tissue could predict the risk of lymph node metastasis [17]. Circulating miR-205 and let-7f together are diagnostic biomarkers for ovarian cancer [18]. Aushev et al. revealed that the level of plasma miR-205 strikingly decreased in patients after removal of lung squamous cell carcinoma [19]. We also assessed whether the miR-205 expression level could function as a tumor marker to distinguish advanced stage from early stage, metastatic cervical cancer from non-metastatic samples and poorly differentiated tumors from differentiated tumors. We found serum miR-205 was a valuable biomarker for cervical cancer patients.

Metastatic spread to regional lymph nodes is considered the most important prognostic factor in patients with cervical cancer [20]. Currently, serum miRNAs have been used to identify lymph node metastasis (LNM) in cervical cancer patients. Zhao et al. reported that miR-20a might be a potential biomarker for detecting the lymph node status of cervical cancer patients, with an AUC of 0.734, a sensitivity of 75% and a specificity of 72.5% [21]. The comprehensive set of serum miRNAs (miR-1246, miR-20a, miR-2392, miR-3147, miR-3162-5p and miR-4484) have great potential to serve as potential biomarkers for LNM in early-stage squamous cell carcinoma, with an AUC of 0.992, a sensitivity of 0.967 and a specificity of 0.950 [22]. In this study, our analysis suggested that serum miR-205 might be a novel biomarker for detecting LNM in cervical cancer patients. Our data also revealed that high levels of serum miR-205 and LNM are independent predictors of poor prognosis. Therefore, as a potential prognostic biomarker in cervical cancer, the use of miR-205 might improve patients’ risk stratification and guide their treatment.

## Conclusions

We observed that serum miR-205 levels could distinguish patients with cervical cancer from healthy controls. The concentration of circulating miR-205 may be an important blood biomarker for cervical cancer screening and represents a potentially useful biomarker for disease progression. The present data suggested that miR-205 might be a valuable circulating marker for cervical cancer with the potential to be translated into clinical applications.

## Methods

### Participants

Cervical cancer patients who underwent surgery between January 2008 and August 2012 in Jiangsu Province hospital on Integration of Chinese and Western Medicine were enrolled in the present study. No patients had preoperative chemotherapy, radiotherapy or other treatment history or other inflammatory diseases. The 60 enrolled patients were aged from 34 to 71 years, with a median age of 51 years. The pathological diagnosis of all 60 cervical cancer patients was cervical squamous cell carcinoma. The 60 control subjects were age-matched, healthy volunteers with no current or previous malignancy. All enrolled individuals gave their informed consent, and the Ethical and Scientific Committees of the hospital approved this study. The degree of differentiation was well to moderately differentiated in 27 cases, and poorly differentiated in 33 cases. Thirty-eight cases had lymph node metastases, while 22 cases did not have lymph node metastases. The clinical stage determined was according to the International League of Gynecology and Obstetrics (FIGO, 2009). Twenty-six stage Ib ~ IIa cases were grouped as early stage, and a total of 34 stage IIb ~ IIIa cases were grouped as late stage. The clinicopathological information of the patients was summarized in Table 1.

### Serum collection

Blood samples were collected in red tiger-top gel separator tubes (Thermo Fisher Scientific Inc., Waltham, MA, USA) from patients or healthy donors. All samples were processed within 2–5 h after collection as follows: the serum was separated by centrifugation at 1,200 × g at 4°C for 20 min and passed through a 13-mm serum filter (Thermo Fisher Scientific Inc.). Serum was divided into aliquots and flash frozen at −80°C until total RNA isolation.

### Tissue samples

The non-tumor counterparts were obtained from a section of the resected specimen at the farthest distance from tumor (>2 cm from the tumor). Tumor and adjacent non-tumor samples were collected at the time of the curative surgery. Resected specimens were routinely processed for histopathological assessment. Each cervical tissue sample (approximately 100 mg) was used for microRNA measurement.

### MiRNA extraction and Quantitative real-time polymerase chain reaction (qPCR)

The Trizol reagent (Invitrogen, Carlsbad, CA, USA) was used to extract total RNA from tissue, according to the manufacturer’s instructions. The miRNeasy Serum/Plasma Kit (Qiagen, Valencia, CA, USA) was used to purify RNA from serum samples according to the manufacture’s instructions. The yields of total RNA were 150–300 ng per 400 μl of serum. For qPCR, miRNA-specific TaqMan MicroRNA Assays (Applied Biosystems, Foster City, CA, USA) for miR-16 (reference miR for serum), U6 (reference miR for tissue) and miR-205 were performed, as described by the manufacturer. Briefly, 100 ng of total RNA was reverse transcribed using primers specific to each miRNA target, followed by real-time PCR using an Applied Biosystems 7000 Sequence Detection System.

The average expression levels of miR-205 were normalized against miR-16 or U6 using the 2^-ΔCt^ method. Differences between the groups were presented as ΔCt, indicating the difference between the Ct value of miR-205 and the Ct value of miR-16 or U6. To ensure consistent measurements throughout all assays, for each PCR amplification reaction, three independent RNA samples were loaded as internal controls to account for any plate-to-plate variation, and the results from each plate were normalized against internal normalization controls.

### Statistical analyses

Statistical analyses were performed using statistical analysis software SPSS 13.0. Data were expressed as the mean ± SD. Analysis of variance (ANOVA) was used to determine the statistical differences among the groups. The Kaplan-Meier method was used to estimate survival rates. A multivariate analysis of the independent prognostic factors was conducted using the Cox proportional hazards model. A receiver operating characteristic (ROC) curve was also constructed to evaluate the specificity and sensitivity of distinguishing advanced stage from early stage, metastatic cervical cancer from non-metastatic samples, and poorly differentiated tumors from differentiated tumors by miR-205 expression levels. A *P* value < 0.05 was deemed significant. A *P* value < 0.01 was deemed highly significant.

## Competing interests

The authors declare that they have no competing interests.

## Authors’ contributions

QM carried out the molecular genetic studies and provided conception and design. GW and SW participated in data acquisition. WY participated in the sequence alignment. JZ contributed to statistical analysis. XY drafted the manuscript and participated in data analysis and interpretation. All authors read and approved the final manuscript.
